# Co-immunization with HIV Env DNA and protein elicit long-lasting strong cellular and humoral immune responses

**DOI:** 10.1186/1742-4690-9-S2-P340

**Published:** 2012-09-13

**Authors:** J Li, A Valentin, V Kulkarni, C Alicea, R Kelly Beach, M Rosati, R Jalah, S Reed, BK Felber, GN Pavlakis

**Affiliations:** 1 Frederick National Laboratory for Cancer Research, Frederick, MD, USA; 2 Infectious Disease Research Institute, Seattle, WA, USA

## Background

We have previously reported that potent, long-lasting HIV-1 Env-specific cell-mediated immune responses could be elicited in rhesus macaques and mice using plasmids encoding env DNA as the immunogen. Subsequent experiments showed that combination of DNA and protein in the form of inactivated virus particles provided significant protection from infection and high viremia. We examine a vaccine platform combining DNA and recombinant Env protein co-immunization at the same time to generate both strong cellular and humoral immune responses.

## Methods

Mice or macaques were immunized with HIV env gp120 DNA vaccine and/or purified gp120 protein from clade B or clade C isolates. Mice were immunized twice at 4 weeks interval with DNA only, protein only formulated in EM005 adjuvant, or DNA&protein/EM005. Macaques were immunized twice at 4 weeks interval with DNA only, DNA&protein, DNA&protein/EM005.

## Results

DNA&protein co-immunization enhances the Ab responses compared with DNA or protein only in mice. DNA&protein co-immunization generated similar levels of cellular immune responses compared to mice immunized with DNA only but those levels were significantly higher than those obtained in mice immunized with protein only. The establishment of a mouse model that gives similar results with the macaque model enhances our ability to test many variations and optimize the vaccine. Importantly, in macaques this strategy elicited higher binding and neutralizing Ab responses than DNA only and the neutralizing Abs showed broad activity. The presence of the EM005 adjuvant further enhanced the Ab responses. These responses were correlated with the up-regulated activation of dendritic cells by EM005. The longevity of the Ab response was superior.

## Conclusion

The strategy of DNA and protein co-immunization has potential for development as a prophylactic HIV-1 vaccine. Our challenge studies show that DNA and protein co-immunized animals developing long-lasting Ab titers were protected from infection.

